# Detection challenges in quantitative polymer analysis by liquid chromatography

**DOI:** 10.1002/jssc.202000768

**Published:** 2020-10-04

**Authors:** Wouter C. Knol, Bob W. J. Pirok, Ron A. H. Peters

**Affiliations:** ^1^ Analytical Chemistry Group van't Hoff Institute for Molecular Sciences (HIMS) Faculty of Science University of Amsterdam Amsterdam The Netherlands; ^2^ Centre for Analytical Sciences Amsterdam Amsterdam The Netherlands; ^3^ DSM Resins & Functional Materials Analytical Technology Centre Waalwijk The Netherlands

**Keywords:** detection, liquid chromatography, polymers, quantification

## Abstract

Accurate quantification of polymer distributions is one of the main challenges in polymer analysis by liquid chromatography. The response of contemporary detectors is typically influenced by compositional features such as molecular weight, chain composition, end groups, and branching. This renders the accurate quantification of complex polymers of which there are no standards available, extremely challenging. Moreover, any (programmed) change in mobile‐phase composition may further limit the applicability of detection techniques. Current methods often rely on refractive index detection, which is not accurate when dealing with complex samples as the refractive‐index increment is often unknown. We review current and emerging detection methods in liquid chromatography with the aim of identifying detectors, which can be applied to the quantitative analysis of complex polymers.

**Article Related Abbreviations**
APCI: atmospheric pressure chemical ionization
APPI: atmospheric pressure photoionization chemical ionization
CAD: charged aerosol detector
CCD: chemical composition distribution
CDMS: charge detection mass spectrometry
CNLSD: condensation nucleation light scattering detector/detection
DLS: dynamic light scattering
ELSD: evaporative light scattering detector/detection
FID: flame ionization detector
ICP: inductively coupled plasma
LAC: liquid adsorption chromatography
LCCC: liquid chromatography at critical conditions
LS: light scattering
MALS: multiangle light scattering
MW: molecular weight
MWD: molecular weight distribution
RID: refractive index detector/detection

## 1 INTRODUCTION

Synthetic polymers are essential to daily life, with a wide number of applications, ranging from various coatings, (biocompatible) medical materials, food packaging to automotive and aerospace materials, and cosmetics. A polymer is not one molecule with a defined structure, but rather a collection of molecules that can feature distributions in molecular size, chemical composition, functional groups, end‐groups, branching, etc. Complex copolymers feature multiple distributions such as molecular weight (MW), chemical composition, and end groups. The final material properties of a polymeric material, and thus the application window, are largely determined by these distributions in molecular structure. Therefore, the determination of the polymer structure is essential to understand material behavior by elucidating the relation between the molecular structure and the material properties.

LC‐based methods are important for the qualitative and quantitative determination of the molecular structure of polymers and their respective distributions. With the application of LC, polymers are typically separated by chemical composition, molecular weight, end‐groups, branching, and/or a combination of these. While LC is a powerful tool in the analysis of polymers, there are some inherent challenges to polymer analysis by LC.

The accurate quantification of the distributions is one of the main challenges in polymer analysis by LC. This is a crucial aspect to the analysis of all compounds but in the case of polymer analysis, accurate quantification is even more complicated. One reason is the limited applicability of the detectors available. As most polymers are not, or poorly UV active, traditional UV/VIS detection is not generally applicable. MS has rapidly become popular [1], but the quantitative application of MS to polymer characterization is rather challenging (if possible at all). The high molecular weight of most polymers complicates their analysis by MS, and the complexity of the spectrum increases due to the different distributions in combination with multiple charge states [1, 2]. Detectors such as refractive index detector (RID) and the evaporative light scattering detector (ELSD) are often applied in polymer LC. While both detectors can be quantitative, meaning that their response is related to the concentration or total mass of the analyte, accurate quantification of complex polymers is very challenging. The reason for this is the strong dependence of the response on the LC conditions and the composition of the eluting polymer fractions (which is often not known), in combination with the absence of well‐defined polymer standards.

To properly understand the issue at hand, it is important to clearly distinguish different detector properties. The first main distinguishing factor between detectors is the kind of information they provide, quantitative and/or qualitative data. A further distinction that can be made is between selective and universal detectors. A selective detector specifically detects a single group (such as a monomeric unit) of a polymer, whereas a universal detector, by definition, detects all eluting polymers. When quantifying polymers, one desires a detector that is quantitative but also universal, since ideally all eluting polymers are detected. This would render detectors such as the RID ideal, but while this detector is generally referred to as a universal detector as it detects most analytes, its response factor is highly dependent on the chemical composition of both the eluent as the eluting polymer. As the chemical composition, and especially the change of chemical composition across the elution profile is not known, this renders the quantification of copolymers with RID very challenging. Therefore, one would ideally desire not just a universal detector but rather one that shows a universal (absolute) response, meaning that regardless of chemical composition of both the eluting polymer and the eluent, the detector features the same response per mass or concentration of the polymer.

Such a detector is currently not available that greatly limits quantitative polymer analysis by LC. Some detectors such as the ELSD and the charged aerosol detector (CAD) have a response factor that approaches a universal response [3, 4, 5]. However, these detectors suffer from other quantification challenges, such as the nonlinear response, but more important, their response factors depend strongly on the eluent composition [6, 7]. Another continuous field of research is hyphenation of LC to detectors such as NMR and IR spectroscopy, or to multiple detectors in tandem [8]. These methods can obtain valuable information on chemical distributions in the sample, and even quantitative information taking several conditions into account. While LC hyphenation with NMR and/or IR spectroscopy in theory forms ideal combinations for qualitative and quantitative evaluation of polymer distributions, their use is still not widespread due to several practical and fundamental limitations.

**FIGURE 1:**
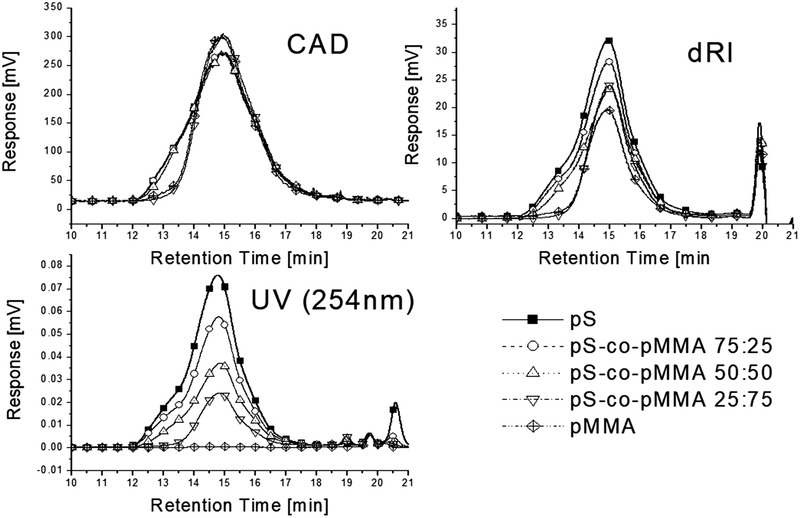
Detection of styrene‐ methyl methacrylate copolymers with various styrene/methyl methacrylate ratios; comparable amounts were injected, chromatograms are not normalized, as detected with different detectors. Note dRI stands for differential refractive index (detection) otherwise referred to as RID in this paper. Reprinted with permission [9], copyright 2017 John Wiley & Sons, Inc

To illustrate the challenges faced in detection, detection with UV/VIS, RID, and CAD SEC of various random styrene‐methyl methacrylate copolymers with different styrene/methyl methacrylate ratios is outlined in Figure 1. A constant sample mass is injected, as can be seen the area differs significantly based on the chemical composition of the sample for the UV/VIS and RID detector.

For the development and analysis of modern materials, which often comprises highly complex polymers, the availability of a quantitative detector is indispensable and arguably a significant bottleneck. It is therefore all the more surprising that this challenge, to the best of our knowledge, has not been covered for polymers in literature to this date. Zhang et al. recently highlighted the general need for universal detectors for analytical separations [10]. A quantitative detector that shows a universal response to all constituents of complex copolymers does currently not exist. Either they do not feature a universal response to all polymers or they require extensive calibration, or optimization for quantification.

In this review, these shortcomings will be addressed and a complete overview of the strengths and limitations of current detection approaches in polymer analysis will be established. Moreover, a number of interesting applications is critically reviewed, with a focus on quantitative detection of complex copolymers. Finally, the recent developments related to LC detectors will be discussed.

## 2 BRIEF INTRODUCTION TO POLYMER ANALYSIS BY LC

Polymer analysis by LC can be performed under a variety of conditions and modes, which dictate the information acquired from the separation. By far one of the most applied separation modes in polymer analysis is SEC, which is a well‐established method for the determination of the molecular weight distribution (MWD) [11]. When applying SEC, molecules are separated by their hydrodynamic radius in solution; larger polymers are excluded from a larger fraction of the porous column packing and thus elute earlier [2]. Other commonly applied separation modes are liquid adsorption chromatography (LAC) and LC at critical conditions (LCCC). While SEC is typically operated in so‐called strong eluents to avoid any interaction with column material, these separation modes operate in weaker eluents, allowing for interaction with the column material resulting in separations based on the chemical structure of the polymer [12]. In LAC, the polymers interact with the stationary phase of the column, thus typically yielding more retention with increasing molecular weight. Separations, in both normal and reversed phase mode, under LAC conditions are often used to examine the chemical composition distribution (CCD) of the sample. In LCCC, there is a balance between SEC and LAC effects, resulting in a separation without MWD effects. This allows for separations based on functionalized end‐groups. New and interesting developments in the field of polymer chromatography are constantly taking place, such as gradient SEC that was recently introduced by Schollenberger et al. [13]. Gradient separations in the LAC regime have recently gained increased importance and thus popularity [12]. However, gradient separations render quantitative analysis more challenging than separations under isocratic conditions, since the response of many detectors is largely influenced by changes in eluent composition. To maximize the value of these developments, absolute quantitative (universal) detection for LC on polymers is needed under both isocratic and gradient conditions.

**FIGURE 2:**
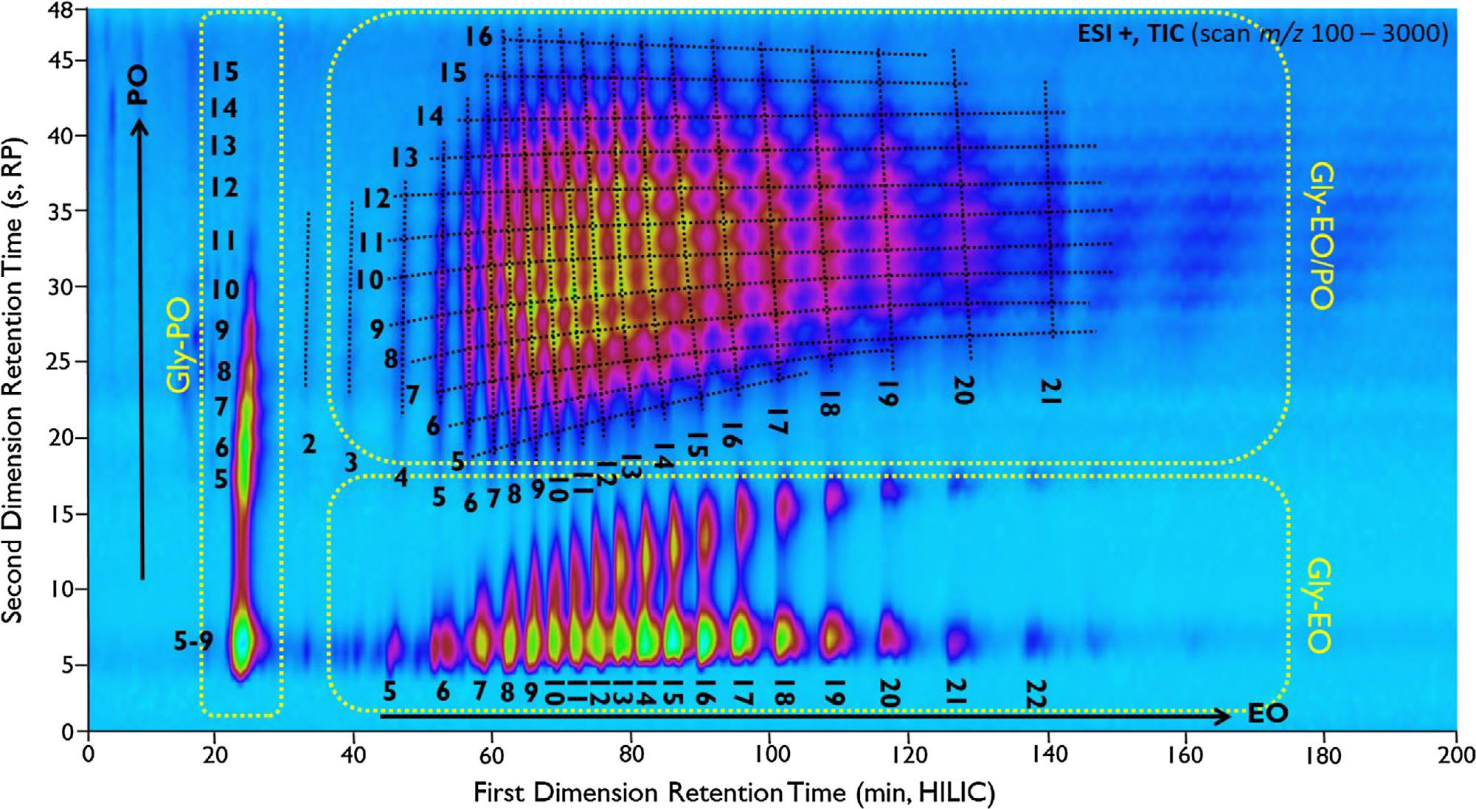
Example of a separation of complex polyether polyols with LC × LC by Groeneveld et al. showing a clear structure based on the number of ethylene oxide/propylene oxide units in the polymer. Reprinted from [16], copyright 2018, with permission from Elsevier

Especially in the field of LC× LC there are many new and exciting developments related to polymer separations [14]. Since polymers typically have a low sample dimensionality, meaning that a low number of variables must be described to identify/characterize a sample, [15] it is possible to obtain structured 2D‐chromatograms. A demonstration of a highly structured 2D‐chromatogram by Groeneveld et al. is illustrated in Figure 2 [16].

Readers interested in more in‐depth reviews on polymer separations are referred to [12, 14, 17, 18]. Overall, we can see a clear shift in the field of polymer chromatography with increasing application of multidimensional separations.

## 3 DETECTORS IN POLYMER LC AND THEIR STRENGTHS AND WEAKNESSES

### 3.1 Summary of commonly used detectors in polymer LC

The ideal detector for quantification in polymer LC has a universal response to all polymers (a response that is independent of the physicochemical properties of the polymer of interest). Additionally, the detector should be applicable regardless of LC conditions (gradient, eluent composition). In Table 1, the features of detectors used in LC of polymers are described, as well as the information provided by the detector on the sample. Additionally, some selected applications are presented per detection principle to illustrate the possible uses of the detector.

**TABLE 1:** Overview of LC detectors and their properties; note that colors indicate suitability as a quantitative detector in polymer separations [19, 20, 21, 22, 23, 24, 25, 26, 27, 28, 29, 30, 31, 32, 33, 34, 35, 36, 37, 38, 39, 40, 41, 42, 43, 44]

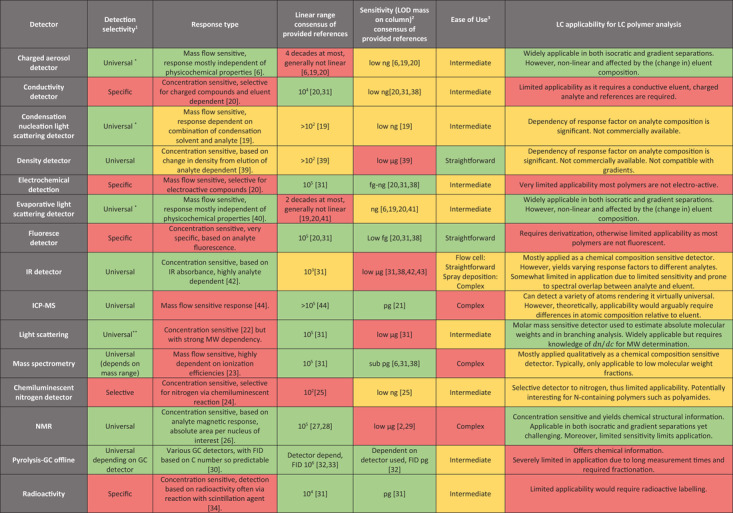		
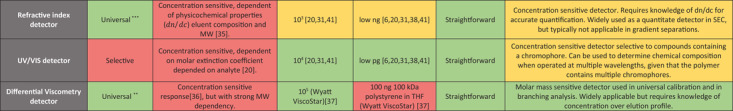		

^1^ Ability to universally detect different analytes rather than featuring a similar response factor.

^2^ It should be noted that the LOD for many detectors is dependent on the analyte, especially for spectroscopic detectors therefore the reported values are only indications.

^3^ It should be noted that the ease of use is inherently a subjective value where provided based on authors experiences and literature.

* For aerosol detectors a significant difference in evaporation temperature should be present between the analyte and eluent; so, no inorganic buffers can be used.

** If compounds are of sufficient MW.

*** If refractive index of analyte differs from the eluents.

### 3.2 Refractive‐index detection

The RID measures the refractive index change of the eluting polymer fraction compared to the eluent. RID is commonly considered as a universal detector for isocratic LC separations of polymers, such as SEC, as it yields a response for most polymers. RID has several advantages as a concentration sensitive detector, such as its ease of use, and straightforward operating principle, although it should be noted that the detector is prone to baseline instability [45]. As can be seen in Table 1, the detector features reasonable sensitivity and linearity compared to other detectors.

The response function of an RID detector can be defined as follows:

(1) $$\begin{array}{c} S_{\mathrm{RID}}=K_{\mathrm{RID}}\times c\times \frac{dn}{dc} \end{array}$$

where *S*_RID_ represents the detector output signal, *c* is the concentration, and *K*_RID_ is an instrument specific parameter. The dn/dc can be defined as:

(2) $$\begin{array}{c} \;\frac{dn}{dc}=\;\lim_{c\;\to \;0}\frac{n-\;n_{0}\;}{c}\; \end{array}$$

where *n* is the measured refractive index and *n*_0_ is the refractive index of the eluent. The dn/dc is defined as the refractive addition index of the polymer at infinite dilution [35]. It should be noted that the dn/dc is dependent on the molecular weight in the oligomeric region but is typically constant above ±5 kDa [46]. The refractive index of the polymer must be different than that of the eluent; iso‐refractive polymers cannot be detected (a well‐known example of an iso‐refractive system is poly‐dimethylsiloxane in THF) [47]. The RID detector is generally not compatible with gradient elution as the response of the detector is heavily influenced by changes in the eluent composition. Although it should be noted that gradient separations are possible if the refractive index of the eluents is nearly identical or adjusted to be identical with an additive [48]. If the chemical composition of the polymer drifts across the MWD, it is also not possible to accurately quantify these copolymers with a RID. If the composition drift is known, the dn/dc can however be predicted for a copolymer as the sum of the dn/dc of the individual polymers multiplied by their respective weight fraction as demonstrated by Laguna et al. [49]. In practice, however, the exact composition and the composition drift as a function of the molecular weight of copolymers are often unknown. Taken in account its limitations, RID is commonly used in SEC to measure the concentration profile of homopolymers in combination with external calibration. They are also used as concentration detectors in combination with online multiangle static light scattering (MALS) and differential viscometry (DV) or dynamic light scattering (DLS) for absolute molecular weight and branching determination. Only if the dn/dc per slice is known (and thus the copolymer composition per MWD), this will result in accurate absolute MWD value. An example of this is given by Haidar et al., who corrected the dn/dc based on the composition across an SEC separation of a styrene‐methyl methacrylate copolymer, using a combination of RID and UV/VIS detection [50]. To summarize, RID can be used as quantitative detector in case the exact dn/dc is known at each point of the separation, which is often not feasible in practice.

As stated above, the RID is typically not gradient compatible, however recent work by Wade et al. showed a novel RID detector based on mirroring resonator arrays, which was compatible with gradient elution since it featured a larger dynamic range [51]. Mordan et al. recently also applied its application in gradient polymer separation [52]. For an in‐depth explanation of the working mechanism of these type of detectors, the reader is referred to ref. [51]. The same technology was also applied for refractive index detection in capillary electrophoresis [53]. Mordan et al. explored the use of this type of detector in SEC separations of polystyrene; the determined polydispersities were in good agreement with conventional detectors but featured a significantly lower S/N [54]. Additionally, an effect of the molecular weight on the sensitivity of the detector was observed. Nevertheless, microring‐resonator‐based detectors are promising options for the application of RID in gradient separations, although their sensitivity is currently still lacking.

### 3.3 UV/VIS detection

In general, UV/VIS detection is by far the most popular detector in LC [20]. The detector measures the light absorbed by the analyte at a selected wavelength or a range of wavelengths in the case of a photo diode array detector [2]. As seen in Table 1, the detector features good sensitivity and linearity compared to other detectors. Furthermore, it is a robust detector that requires little maintenance and is easy in use. With many polymers of interest not featuring a chromophore group, the applicability of UV/VIS spectrophotometry to the analysis of polymers is rather limited. Therefore, UV/VIS spectrophotometry is mostly applied as a selective detector in polymer analysis to study the composition fractions of UV‐active monomers, such as styrene, in copolymers [49, 50, 55, 56]. Alternatively, derivatization of reactive groups with UV active derivatives can be used to monitor the distribution of the derivatized group. Brooijmans et al. demonstrated this on acrylate copolymers containing methacrylic acid monomeric units. By derivatizing of methacrylic acid with UV‐active phenacyl bromide, the fraction of methacrylic acid across the MWD could be quantified in various copolymers with SEC‐UV‐RID [57]. Besides derivatization so‐called indirect detection can be applied in which nonabsorbing analytes are detected indirectly by displacement of an absorbing probe, which is added to the eluent [58]. This principle can be applied to multiple detection mechanisms, but in practice is rarely applied to polymers. However, indirect UV detection has been used in CE separations of polymers [59]. Hoeylandt et al. applied a diode‐array detector to determine the chemical distributions from SEC traces of mixtures of polystyrene, poly(ethoxyethyl acrylate), and polymethyl methacrylate [60]. Data processing was performed by a chemometric approach using all UV/VIS spectra across the chromatogram instead of defined wavelengths. By applying a multivariate curve resolution‐alternating least squares method, the signals of the individual homo polymers could be deconvoluted and quantified. Such approaches can only be applied to polymers that are composed of UV/VIS sensitive monomers. Polyethers such as polyethylene glycol and polytetrahydrofuran have no significant UV/VIS response, which renders the approach inapplicable to these samples. Furthermore, the use of multiple monomers (*n* ≥ 3) in copolymers complicates this approach, especially if they feature similar UV/VIS spectra. And as a final remark, the UV/VIS response of each monomer should be known beforehand.

### 3.4 Aerosol‐based detectors

Aerosol‐based detectors such as the ELSD have gained increased popularity the last decades [61], as they are able to measure under gradient conditions and (due to more uniform response factors) have less dependency on the chemical composition drift. These detectors are considered to be universal since they detect any compound with a higher boiling point than the eluent, which is evaporated before detection. While these detectors can be used quantitatively, they lack a wide linear dynamic range typically showing an exponential response function [61]. Briefly summarized, the ELSD functions in the following manner; the eluent is nebulized and subsequently evaporated transforming nonvolatile species into dried particles, which are detected by light scattering detection mainly based on Mie scattering [62]. Its response is affected by the dried particle size that typically differs as a function of concentration rendering it nonlinear [63].

The CAD is comparable to the ELSD in operating principle, although it is less widely applied. The response of both detectors can be described with the same response function (Equation 3). Contrary to ELSD, CAD analytes are detected based on their charge induced by collision with a charged gas [6]. As mentioned above, the main drawback of the ELSD and CAD is their nonlinearity, otherwise featuring reasonable sensitivity [6, 19, 64]. The nonlinearity is problematic when, determining MWDs with SEC because the peak shape will not accurately describe the concentration and thus result in errors [5].

Generally, the nonlinear response of the ELSD and CAD is expressed as an exponential relationship.

(3) $$A=a\cdot m^{b}$$

where *m* is the injected mass and *a* and *b* are instrument parameters. The value of *b* typically differs between 0.9 and 2.0 for the ELSD. If values are close to 1, linear calibration lines can be constructed. A number of efforts have been made to improve the linear response of these detectors by mathematical corrections [62]. An example of a commonly used linearization method is given as:

(4) logA=b·logm+loga

Alternatively, the peak area can be raised to the power 1/*b* to yield a linear relation between peak area and injected mass. This does however require previous determination of *b* and proper baseline correction before application.

(5) $$A^{1/b}=a^{1/b}\cdot m=k\cdot m$$

Mengerink et al. proposed the inclusion of a factor correcting for differences in peak widths to improve the quantitative accuracy of the ELSD [65]. The linearization method in Equation 5 was applied by Boborodea et al. to perform a segmental correction across the whole chromatogram of data acquired with a high‐temperature ELSD signal after baseline correction. Using this method, linear calibration lines for polystyrene and polyethylene were acquired [5]. A similar method using segmental correction was applied by Biedermann et al. to accurately quantify low molecular weight polyadipate fractions [66].

**FIGURE 3:**
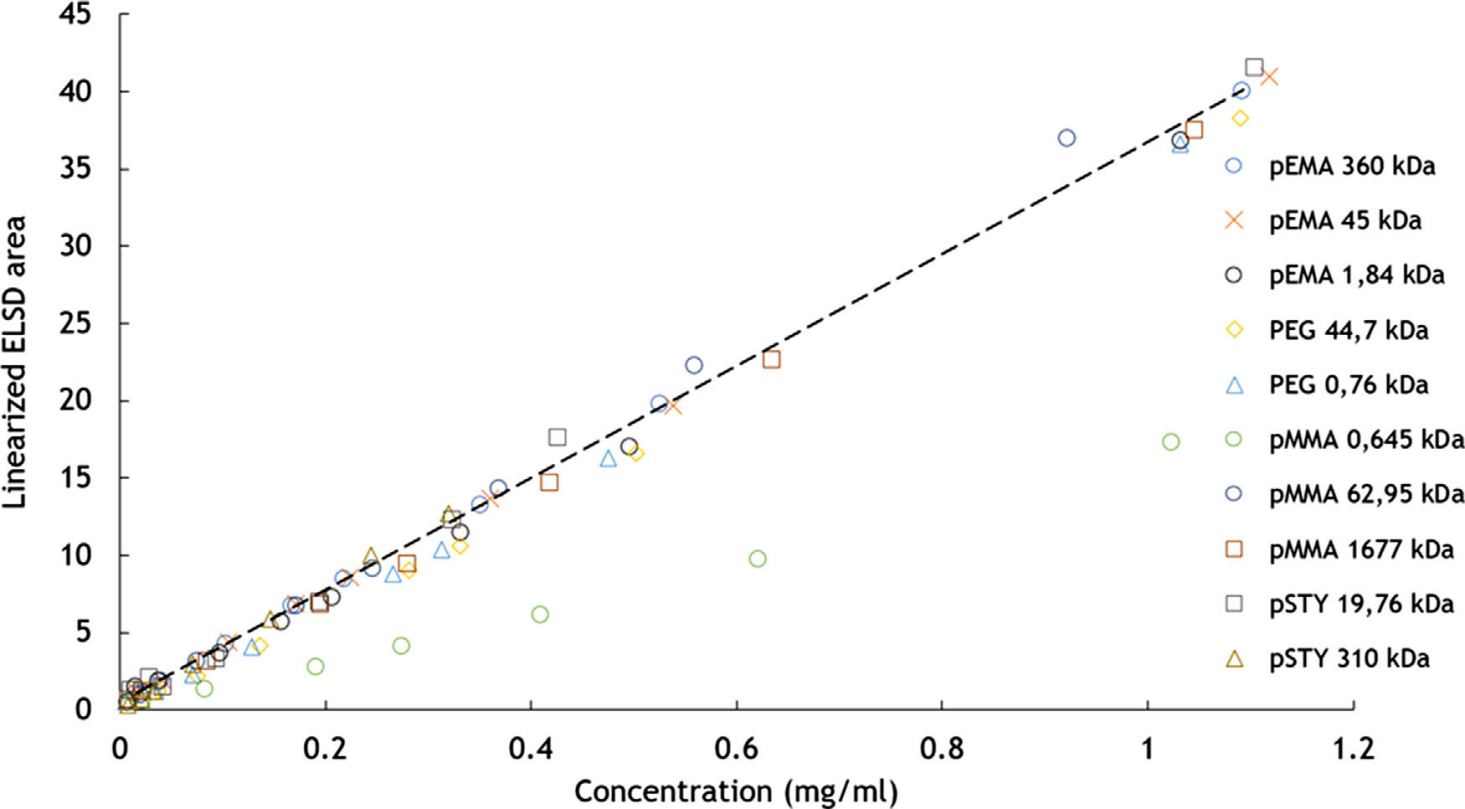
Graph by Brooijmans et al. showing a linear relation after correction and similar response functions for a variety of polymer samples of different molecular weight distributions. Reprinted from [69], copyright 2020, with permission from Elsevier

Arndt et al. published a comprehensive overview of ELSD applications in polymer analysis in 2013 [40]. Most applications reported an exponential response curve, while a number reported a linear or sigmoidal calibration line. Regarding the universal response of the ELSD for different compound classes, a review specifically addressing this point arrived to no clear conclusion if individual calibration lines need to be constructed for every analyte [67]. The detectors mass response might not be identical for all compounds but is generally in good agreement, therefore, the use of compound specific calibration or individual response factors would rely on the required accuracy. Regarding polymers specifically, Arndt et al. concluded based on the reviewed literature that there is no clear consensus on the effect of the chemical composition on the response. Most authors found no molecular weight influence on the detector response [40]. Recently, a new type of ELSD was introduced that allows the evaporation of eluents with a higher boiling point, as used in high temperature SEC. This ELSD featured a similar linear response for linear low‐density polyethylene and polystyrene after correction [5, 68]. Recent work by Brooijmans et al. applying this detector demonstrated a linear response after correction as described by Boborodea et al. and similar response functions for a variety of polymers, as can be seen in Figure 3 [69].

While the response of the detector can be linearized using models such as Equation 5, the response is also dependent on the eluent composition. This effect still limits the quantitative applicability of the ELSD and the CAD in gradient elution methods. Efforts have been made into combating this effect by eluent compensation for both ELSD and CAD, where the eluent is adjusted after separation but before detection to keep the eluent composition entering the detector constant [3, 4]. This approach was used by Eckardt et al. to quantify oligomers extracted from a variety of polymers with the CAD [70]. By use of universal response‐based quantification, a variety of oligomers could be quantified, a variation in the response factor of ±20% was found between different oligomers. While the eluent compensation method improves the consistency of the response function for both detectors, it has obvious drawbacks since it requires an extra instrumentation and it dilutes the analyte twofold before detection [3, 4]. Alternatively, efforts have been made to construct a 3D calibration surface of concentration versus response at different eluent compositions to accurately quantify for both ELSD and CAD in gradient LC [7, 71]. Such an approach requires a large amount of calibration measurements that however is not ideal.

Kou et al. compared the CAD to ELSD and RID for the analysis of polyethylene glycol. They found that the CAD was slightly more linear than ELSD and more sensitive than RID [72]. Another comparison was made between CAD and ELSD by Takahashi et al. who analyzed polyethylene glycol of different MWDs with supercritical fluid chromatography coupled to both ELSD and CAD [73]. The sensitivity of the CAD was found to be 10 times higher than that of the ELSD and it featured better reproducibility.

The condensation nucleation light scattering detector (CNLSD) commercialized under the name NQAD also has a similar working principle as the ELSD, but features a condensation chamber where a gas is condensed on the dried particles before light LS detection to render detection more sensitive [19]. The universal response character of the ELSD is lost however in the CNLSD since the response of the analyte is also dependent on the affinity of the condensation gas for the analyte. Koropchak et al. applied the CNLSD and ELSD to various polymers such as polyethylene glycol, polyacrylic acid, and dextran [74]. They found that the CNLSD yielded significantly different response factors per analyte as a function of mass compared to the ELSD, although it was more sensitive and showed a better linear range. Since the CNLSD is no longer commercially available, it will not be discussed in more detail.

In summary, ELSD and CAD are considered as universal mass‐based detectors for nonvolatile compounds. Since their response is nonlinear and influenced by the gradient, their quantitative use is not straightforward. The CNLSD does not feature the same universal response as the ELSD and CAD rendering it a less suited detector for complex copolymers.

### 3.5 Light‐scattering detection

Static light scattering (SLS) detectors measure the time‐average Rayleigh scattering intensity at one or more detection angles. Combined with a concentration‐sensitive detector, SLS is one of the few detection methods allowing absolute molecular weight determination. At multiple angles, SLS experiments also yield information on the size of the analyte [22]. While there are various SLS‐based detection methods, overall SLS detectors feature a relatively large linear range (Table 1) but a low sensitivity. It should, however, be noted that the sensitivity largely depends on the MW of the analyte since SLS detectors are MW‐sensitive detectors.

There are three main types of SLS detectors: (1) the low‐angle SLS detector (typically 15°), (2) the right‐angle SLS detector (typically 90°), and (3) the multiangle SLS detector [22]. SLS detectors feature response factors that can be expressed as shown in the following equation [75]:

(6) $$\frac{K_{i}\cdot c_{i}}{R{\left(\theta \right)}_{i}}=\frac{1}{M_{w,i}\cdot P{\left(\theta \right)}_{i}}+2A_{2,i}\cdot c_{i}+3A_{3,i}\cdot {c_{i}}^{2}+\cdots$$

Here, *i* refers to a specific detector increment, R(θ) is the excess Rayleigh scattering, *c* is the concentration of the analyte, $M_{w}$ is the molecular weight, P(θ) is the angular dependence of the light, and *A*_2__,_ *A*_3_ are the second and third virial coefficient, respectively [2]. For concentrations at near infinite dilution, typically used in SEC, the effect of the second and higher order virial coefficients can be assumed to be negligible. At low angles, P(θ) is close to 1 [75]. Here, *K* is a constant at a given dn/dc:

(7) $$\begin{array}{c} K=\frac{4\pi^{2}{\left(\frac{dn}{dc}\right)}^{2}}{\lambda^{4}N_{A}} \end{array}$$

where $N_{A}$ is Avogadro's number and λ is the wavelength of the scattered light. It should be noted that, since the intensity of the SLS detector depends on the MW, the sensitivity for low MW compounds is rather low, thus resulting in a need for high concentrations of lower MW compounds, which can result in issues with chromatographic peak tailing and the need to take the virial coefficients into account [46].

SLS detection has seen wide application in LC separations of polymers to monitor the molecular weight or size (radius of gyration, Rg) across the elution profile. The main applications are absolute MWD determination [50] and branching analysis [76, 77, 78]. A more recent development in the field is the introduction of DLS, which monitors the time‐dependent fluctuations of the light scattering caused by Brownian motion that can be related to the diffusion coefficient from which the hydrodynamic radius (Rh) can be determined [46]. Combination of SLS and DLS renders both Rg and Rh, the ratio of which is indicative of polymer shape in solution.

While SLS detection is applied to determine the molecular weight, it is severely limited in its application to complex samples since it requires knowledge of the dn/dc, which is not trivial to determine for complex samples (Section 4.1). Lavric et al. recently proposed an alternative approach to apply SLS without a known analyte dn/dc [79]. This method relies on calibration with a standard of known molecular weight but does not require the dn/dc of the analyte or calibrant. When applied to a variety of polymers, the authors observed an average deviation of less than 10% compared to MW values determined with the dn/dc. Furthermore, knowledge of concentration at each point of elution is required, which is challenging to determine accurately for complex samples. Another factor complicating the application of LS is that co‐elution of analytes with different dn/dc values can result in errors in the determined MW [75]. Furthermore, analytes (such as lignins) can show fluorescence leading to erroneous MW determination; this effect can be reduced however by the installation of fluorescence filters [80]. Overall, LS is the prime method to determine the absolute MWD, but it fully relies on the concentration determination, which is often challenging for complex copolymers.

### 3.6 Mass spectrometric detection

MS transforms analytes into gas‐phase ions and separates the formed ions according to their *m/z* (mass relative to charge). MS detectors generally feature a large dynamic range (∼10^5^) and a relatively high sensitivity (Table 1), rendering them the detector of choice in many applications. MS is widely applied in polymer analysis for the investigation of various sample characteristic such as the end‐group distribution, the chemical composition, and the MWD [81]. Besides since it can be used to determine the absolute mass of analytes, it finds use in the absolute mass calibration of SEC [82].

ESI is one of the most applied ionization techniques to introduce synthetic polymers in the MS [1]. In ESI, the LC effluent is sprayed by means of a potential difference. In the interface, the eluent is evaporated, allowing charges to be transferred to the analytes from the eluent. ESI can typically only be applied to the analysis of (moderately) polar polymers, since it is a relatively soft ionization method [83]. However, addition of low concentrations of salts/buffer to the mobile phase enables the analysis of less polar polymers, such as polystyrene, with ESI‐MS [84, 85]. While ESI is typically incompatible with non‐ or low‐polar solvents, limiting its application to polymer analysis, this can be improved by an additional spray with a polar solvent and possibly a dopant [86]. Another challenge encountered when applying ESI in polymer LC is that ESI typically yields multiple charge states of each polymer species resulting in a very complex spectrum [83]. As the ESI‐MS is limited to MWs of a few kilodalton [2], combined with the complex MS spectra for complex copolymers, the applicability of ESI‐MS for broadly distributed or high molecular weight polymers is very limited. Nevertheless, ESI‐MS has been successfully applied to study the exact MW and structure of low MW oligomers [8]. Song et al., for instance, applied LC‐ESI‐MS to study the polymerization mechanisms of poly(n‐butyl acrylates) in different solvents by differentiating the samples based on fragmentation pathways and end‐groups [87]. Hisatomi et al. applied LC‐ESI‐MS to model the relation between retention time and composition for various acrylate copolymers [88]. Recently, Steinkoenig et al. demonstrated that through chlorine attachment and the possible addition of supercharging agents (i.e., sulfolane or propylene carbonate), even medium nonpolar polymers, such as polystyrene, could be analyzed with ESI‐MS [89]. By application of this ionization process, the authors were able to analyze polystyrene and polybutadiene of masses up to 18 and 10 kDa, respectively. Epping et al. recently explored the use of LC‐ESI‐MS in the microstructure determination of copolymers; by combining retention time, chromatographic peak width, and MS information, it was possible to elucidate in depth microstructure and topology information on various glycol and ethoxylate oligomers [90]. Furthermore, by using separation at LCCC conditions coupled to ESI‐MS insight into the MWD, end‐group distribution, CCD, as well as the sequence of ethylene oxide‐propylene oxide copolymers could be obtained [91]. Cramer et al. recently applied a chemometric method aiming to predict the mass spectral counts of various drug monomers, with promising results [92]. While not yet applied to polymers, its application would be interesting since polymers consist of a variety of molecular species of which there are no individual standards available.

Another MS approach for the characterization of polymers is MALDI‐MS [1]. To analyze a compound with MALDI‐MS, the analyte is deposited on a solid substrate with a matrix (typically a small polar molecule with a chromophore) and dried. The mixture of analyte and matrix is excited by a pulse from a laser at a wavelength adsorbed by the matrix, yielding gas‐phase ions from a charge transfer from the matrix to the analyte. A major advantage is that MALDI‐MS mainly yields singly charged ions that results in a simpler and easier to interpret spectrum. Compared to other MS techniques, the accessible mass range is typically higher, which renders MALDI‐MS especially suited for polymer analysis. MALDI‐MS analysis can be applied to a wide range of polymers and analyze polymers with molecular weights in excess of 10^6^ Da [1]. It should however be noted that when analyzing samples with a wide polydispersity >1.2, a significant bias in the determined values will be observed due to different ionization and detection efficiencies over the MWD [1]. It should be noted that besides the MWD, many factors influence the ionization efficiency and that every unique chemical structure will have a different ionization efficiency [93]. Cox et al., for instance, showed a large influence of the end‐group of PS samples on the ionization efficiency [94]. Another downside is that MALDI‐MS is difficult to apply online since the analyte must be dried together with the matrix [83]. Nevertheless, coupling to LC has been performed, typically via an automated offline approach by spotting or spraying the analyte on a MALDI substrate plate. Barqawi et al. applied online MALDI/ESI‐MS coupled to 2D‐LC to monitor the composition and end‐group functionality of α,ω‐telechelic poly(ε‐ caprolactone)s [95]. Trimpin et al. developed a fractionation method to perform automated offline MALDI under solvent‐free conditions [96]. The method was demonstrated on poly(ethylene oxide) samples with different end‐group functionalities and molecular weights. Town et al. applied MALDI‐TOF/TOF on various acrylate polymers and copolymers, and using fragmentation it was possible to distinguish between block and statistical copolymers [97].

Next to ESI and MALDI, alternative interfaces such as atmospheric pressure chemical ionization (APCI) and atmospheric pressure photoionization (APPI) are also applied in polymer analysis [98]. In APCI, a discharge needle is used to ionize analytes, whereas in APPI, UV light is used. A major advantage of these methods is that these allow direct coupling to LC with nonpolar solvents, which are typically incompatible with ESI. These methods also typically yield singly charged ions. Although these interfaces have seen little application in polymer analysis, probably due to their less widespread use and more complex ionization mechanisms, they are considered to be more suitable for the ionizations of nonpolar molecules than ESI. The mass range is limited compared to ESI and MALDI, to <1500 Da, as gas phase introduction is challenging and thermal decomposition is common for larger molecules in APPI and APCI, however APPI and APCI are still options for application in polymer analysis [98, 99, 100, 101].

Another interesting development in polymer MS is charge detection mass spectrometry (CDMS). In CDMS, the mass of analytes is determined directly from the measurement of charge and *m/z*. More details on the working mechanism of CDMS can be found in ref. [102]. The main advantage of CDMS is the high upper mass range (MDa) of the mass analyzer [102]. CDMS was coupled to SEC by Viodé et al. for the absolute mass calibration of high molecular weight polyacrylamide and poly(2‐acrylamido‐2‐methyl‐1‐propanesulfonic acid) standards [103]. The average mass values of 5–6 MDa for polyacrylamide and 2 MDa for poly(2‐acrylamido‐2‐methyl‐1‐propanesulfonic acid) specified by the manufacturer were comparable to values found with both direct‐infusion (6.5 and 2.3 MDa, respectively) and SEC‐CDMS experiments (7 and 3.1 MDa, respectively). It should be noted that MS/MS methods and ion mobility spectrometry are also of interest to polymer characterization but are outside the scope of this review, as these methods are often applied to more in‐depth structure analysis. The interested reader is referred to refs. [104, 105, 106, 107, 108].

Regardless of the ionization method, the main drawback of MS is the limited mass range. Since the ionization efficiency depends on the eluent composition but also of the chemical nature of the copolymers, the response factor differs widely between analytes [1]. This thus renders MS semiquantitative at best in polymer analysis, given that standards are available and that the sample is of low molecular weight. The application of MS in polymer analysis is thus mainly focused on identification of the chemical composition and structure of the copolymers.

### 3.7 NMR spectroscopy detection

NMR spectroscopy is a powerful tool in polymer analysis. NMR spectroscopy is intrinsically quantitative and features a large dynamic range while also yielding information on the chemical structure of the sample. Coupled with LC, NMR spectroscopy can provide quantitative information on a variety of chemical structures such as the chemical composition and the polymer sequence across an elution profile [26]. NMR spectroscopy is, however, difficult to employ as detector in LC due to its cost, but particularly due to its extremely low sensitivity (μg, Table 1) [26, 109].

Since the sensitivity of NMR spectroscopy is inherently low, an averaged spectrum of multiple scans is typically required to achieve sufficient S/N [26]. The frequency at which these scans can be acquired depends on the *T*_1_ relaxation time of the nuclei of interest, since it must be fully relaxed before the next pulse is applied in order to yield a quantitative signal (typically 1–10 s). Since the averaging multiple scans lead to a higher sensitivity, there is thus a trade‐off between the overall analysis time, the S/N, and the density of averaged data points. This can result in a scenario where the separation must be slowed down to acquire spectra of sufficient quality. Alternatively, stop‐flow methods or loop‐based collections systems can be applied to allow for more measurement time. Since deuterated eluents are expensive, experiments are often performed in nondeuterated eluents requiring suppression of the eluent signals. This suppression of the eluent signal leads to missing parts in the spectrum around the suppressed eluent signal, which is a major drawback to LC‐NMR, rendering a variety of eluent polymer combinations incompatible [109].

As the combination of LC and NMR spectroscopy is very powerful with regard to quantification and qualification, quite some research has gone into the application of LC‐NMR in polymer analysis. Krämer et al. applied SEC‐NMR to monitor the chemical heterogeneity of styrene‐ethyl acrylate copolymers as a function of the MWD. The applied method acquired a full NMR spectrum every 40 s, using a 400 MHz instrument, yielding 5–10 data points across the MWD [110]. Kitayama et al. applied LC‐NMR to study the tacticity of poly(ethyl methacrylate)s after LCCC separation using a 750 MHz instrument [111]. Hehn et al. applied LC‐NMR to study the tacticities of poly(methyl methacrylate)s and to monitor their isotope (D vs. H) distribution. Using both ^1^H‐ and ^2^H‐NMR spectroscopy, it was possible to demonstrate that poly(methyl methacrylate) samples could be separated on their isotope distribution in LCCC [112]. Hiller et al. hyphenated NMR spectroscopy to high temperature SEC expanding its applicability to polymers, which are not soluble at low temperatures such as polyolefins [113]. The system was demonstrated on a variety of polyethylene and polymethyl methacrylate homopolymers and copolymers, and the separation was performed at 130°C using 1,2,4‐trichlorobenzene as a solvent. Recent developments have shown that lower field strength NMR instruments might also be applied in LC‐NMR. Höpfner et al. performed LC‐NMR on a low‐resolution (62 MHz) benchtop NMR instrument [114]. The instrument was carefully optimized to yield an optimal S/N, yielding an LOD of 0.36 mg of injected mass for polystyrene.

While LC‐NMR is potentially a promising method in polymer analysis, limitations in sensitivity, solvent suppression, and cost of the high‐sensitivity equipment render its current application limited. Nevertheless, when LC‐NMR is applicable, its ability to perform absolute quantification renders it an extremely useful tool for the characterization of complex polymers.

### 3.8 FTIR detection

The FTIR detector is a detector of interest in polymer analysis as it can detect most polymers and offer information with respect to the chemical structure. The detector features a relatively low sensitivity and an average dynamic range (Table 1). Two main types of LC‐IR detectors exist: the online flow cell detectors, similar in design to UV/VIS detectors, and the evaporative‐solvent interface detectors [42]. The development of the latter was mainly driven by the removal of the background signal caused by the eluent. The eluent is evaporated, and nonvolatile species are deposited on an IR transparent disk and subsequently detected by an IR spectrometer. The quantitative capabilities of these detectors are mainly affected by the quality of the created film [115]. There are a number of strengths and weaknesses for both detectors. Kok et al. compared both detectors and found a better repeatability for the online flow cell type and a higher sensitivity for the evaporative detector [42, 116]. Besides, the evaporative type is more compatible with gradient elution, as the gradient will not affect the measurement. The ease of operation is greater for the flow cell type, as the evaporative type requires careful optimization of evaporation conditions to yield a high‐quality film.

**FIGURE 4:**
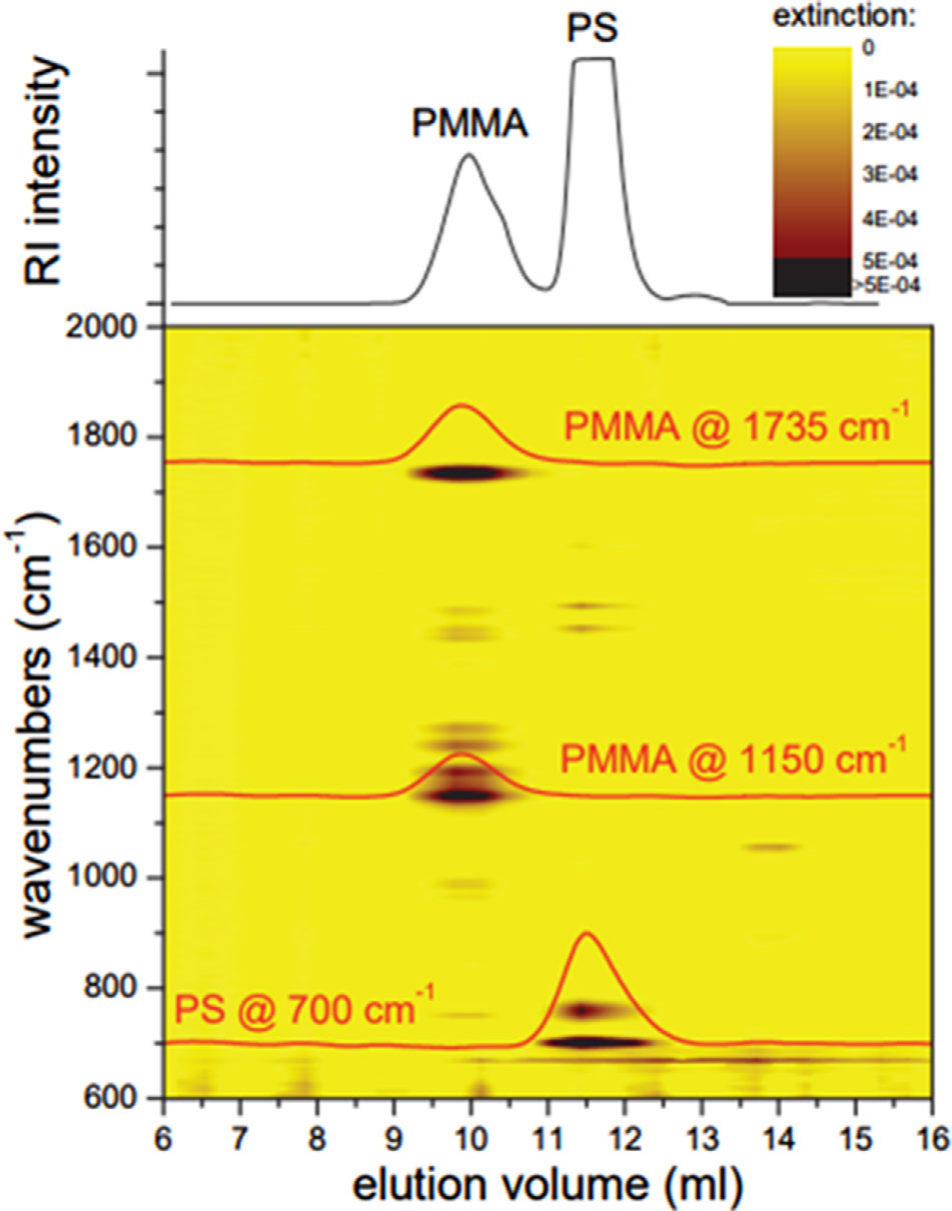
SEC‐IR measurement showing online measured IR spectra of poly(methyl methacrylate) and polystyrene across the elution profile. Reprinted with permission from [122], copyright 2012, WILEY‐VCH Verlag GmbH & Co. KGaA, Weinheim

The flow cell IR detector is often applied to determine short chain branching polyolefin blends where the methylene and methyl band can readily be distinguished in the IR spectra [117, 118, 119, 120]. An in‐depth research on the quantitative aspects was recently published by Frijns‐Bruls et al. [121]. Recently, Beskers et al. improved upon the online flow cell type IR detector by applying a variety of improvements such as an attenuated total reflection‐based flow cell, a quantum detector, and improved detector software. This resulted in an improvement in the S/N for a given measuring time by a factor of 70 000, and an example of the application of the detector is featured in Figure 4 [122].

The same group expanded this work by addition of an additional IR detector in tandem in the form of a quantum cascade laser‐IR spectrometer, which is in essence an IR spectrometer with a high‐intensity laser as a light source. This detector operates at a single narrow wavelength with increased sensitivity and can thus be applied to monitor a specific functional group at lower concentrations. The quantum cascade laser‐IR setup proved to be 3.8 times more sensitive for poly(methyl methacrylate) than the full‐scan mode used in the conventional IR spectroscopy that was connected in tandem [43]. IR detection was also applied in 2D‐LC of styrene‐methacrylate copolymers by Kok et al. [123]. Using the IR detector, changes in the chemical composition of the polymer could be monitored across the elution profile.

While there are some promising recent developments, IR detection suffers some clear drawbacks. Flow cell type detectors feature low sensitivity and are limited in analyte eluent combinations, although they feature good quantitative capabilities. Spray deposition type IR detectors do not suffer from these drawbacks but show relatively poor quantitative capabilities. Overall, monitoring the total concentration of a complex sample with multiple different monomer types by IR detection is still quite challenging as the concentration of all monomers need to be determined individually and summed up. Both types of IR detectors thus feature clear drawbacks as a quantitative detector for industrial samples.

### 3.9 Fluorescence detection

Fluorescence‐based detection is an excellent method for the selective and quantitative detection of fluorescence compounds by LC. Fluorescence detectors feature very high sensitivity (fg range) and a large linear range (10^5^, Table 1), rendering them attractive quantitative detectors when applicable. The application of fluorescence detection to polymer LC is however limited since most polymers are not fluorescent. Nevertheless, a number of groups found applications to polymer analysis, mostly on polystyrene. Biver et al. applied SEC coupled to a fluorescence detector to characterize polystyrene and degraded polyolefins in microplastics found in beach sediments [124]. The method could detect polystyrene and degraded polyolefins and was shown to be linear in ranges from 25 to 5500 and 720 to 7400 mg/L, respectively. Broersen et al. developed a method for the derivatization of polyamide resins with o‐phthalaldehyde before analysis with SEC‐fluorescence detection [125]. While the selectivity of the fluorescence detector renders that it is not applicable to most polymers, it may prove to be interesting for the selective quantification of functional groups after derivatization.

### 3.10 Differential viscometry detection

Differential viscometers are commonly applied in polymer LC, and they typically function by measuring a pressure drop across a capillary versus a reference capillary filled with eluent. These molecular weight sensitive detectors can be applied to determine the degree of branching in combination with other detectors (see Section 4.2) [75]. Also these detectors are more suited for studying the molecular weight and molecular architecture distribution rather than quantitation. The generally lacking sensitivity of these detectors is influenced by a variety of factors including the molecular weight. As a reference, the ViscoStar III viscometer is able to detect 100 kDa polystyrene in tetrahydrofuran across a large dynamic range of (10^5^) but features a limited sensitivity of 0.1 μg [37]. When combining a concentration sensitive detector, such as a RID, with a DV, the intrinsic viscosity [η] can be determined by dividing the specific viscosity measured by the DV with the concentration [36]. Another major use of the DV is universal calibration, allowing MW determination with SEC without compound specific calibration; this can be achieved by the coupling of a concentration‐sensitive detector such as a RID with DV. As discovered by Grubisic et al., a plot of log(M·[η]) versus the retention volume in SEC yields a curve that is identical for most polymers, regardless their chemical composition [126]. This renders the use of a coupled viscometer, especially useful when analyzing samples for which there are no molecular weight standards available [36]. The intrinsic viscosity [η], in turn, is defined as:

(8) $$\begin{array}{c} \left[\eta \right]\equiv \lim_{C\to 0}\frac{0\eta_{sp}}{c} \end{array}$$

where *c* is the concentration of polymer in the near‐infinitely dilute solution. To determine [*η*], a concentration‐sensitive detector (typically a RID) is needed, and the intrinsic viscosity is thus recognized as the ratio of the signal from the viscometer, which measures η_sp_, to that of the concentration‐sensitive detector. This thus requires accurate quantification of the polymer across the elution profile, which is challenging to obtain. In short, the DV is not suitable as a concentration detector due to the strong influences of the MW on the response but is rather used in universal calibration and branching determination.

### 3.11 Conductivity detection

Conductivity detectors measure the electrical resistance of the eluent. The elution of a charged analyte changes the conductivity of the eluent resulting in a detector response. Conductivity detectors typically feature an average sensitivity (ng) and a good linear range (10^4^_,_ Table 1). The analyte must be charged in order to be detected and eluent conductivity is required. Therefore, conductivity detection is generally only applied in aqueous chromatography systems, which limit its application to most forms of polymer LC. Alternatively, indirect detection in conductive solvents can be applied for noncharged analytes [127]. Chaidedgumjorn et al. applied conductivity detection in an aqueous SEC system to determine the molecular weights of per‐O‐sulfonated glycosaminoglycans, achieving LODs of 1–10 ng [128]. Another application by Oudhoff et al. reported the use of contactless conductivity detection in nonaqueous size‐exclusion electrokinetic chromatography of synthetic polymers [129]. In this study, polystyrenes were separated using a dimethylformamide eluent containing LiCl and subsequently detected by conductivity detection. Since the detector is not universal and requires conductive eluents, it is limited in application as a selective detector in aqueous SEC or CE separations of polymers.

### 3.12 Flame ionization detection

The possibility to use the flame ionization detector (FID) as an LC detector is a major advantage, since it features a universal response factor, a large linear dynamic range (10^6^), and high sensitivity (pg, Table 1). Since the FID is sensitive to all organic compounds, direct coupling to LC with organic eluents is likely to overload the detector. Therefore, a variety of approaches has been developed to couple LC to FID; in practice, there are two routes to do so: indirect coupling via pyrolysis‐GC and direct coupling approaches, which do not include a GC separation.

When applying pyrolysis‐GC, the sample is pyrolyzed by heating it to high temperatures in an inert environment, after which the resulting pyrolysate is separated and detected [130]. This allows for the use of, among other GC detectors, the FID. Furthermore, the quantitation of monomers and small oligomers formed during pyrolysis is more straightforward than that of intact complex polymers since they are individual molecules rather than distributions. LC coupling with pyrolysis‐GC‐FID/MS is generally performed offline, which renders it laborious and time consuming. While coupling to MS besides FID has its merits, its quantitative applicability is more challenging since the response factor is less predictable. Some efforts have gone into hyphenation of polymer separations to pyrolysis‐GC. Kaal et al. hyphenated (stop‐flow) SEC to pyrolysis‐GC using a side port syringe as a GC injector [131]. While allowing automated SEC‐pyrolysis‐GC hyphenation, analysis times were significantly longer due to the slow second dimension analysis times. The same approach was later also applied by Brander et al. for the characterization of high MW light stabilizers [132]. Zhu et al. applied an evaporative interface that deposited SEC effluent on a rotating aluminum foil, and sections of the foils were excised and subjected to pyrolysis‐GC [133].

The direct coupling of FID to LC has been studied extensively [134, 135, 136]. The developed hyphenation methods typically collected the LC effluent on a moving belt or wire, which transported the analyte to the FID. A belt‐type detector with a pyrolysis chamber was applied to the detection of polystyrene standards separated by SEC in 1968 by Johnson et al. achieving microgram sensitivity [137]. Other methods to couple LC to FID include direct hyphenation by capillary jet interfaces. A recent review by Becker et al. summarized a variety of approaches to couple FID to LC [33]. It should be noted that most coupling methods yielded a reduced sensitivity compared to GC‐FID typically yielding nanogram‐level sensitivity. Another disadvantage of direct hyphenation methods such as capillary jet interfaces is that they are only compatible with aqueous or low organic eluents. Around 2001, the moving‐belt concept was reintroduced in the form of the UNIMAS detector that used an argon ionization detector, but it was never fully commercialized [138]. More recently, the Solvere™ was developed by Activated Research Company [139]. This detector evaporates the eluent and applies catalysis to transform the analytes into methane, after which the formed methane is detected by FID detection. While not yet commercially globally available, the concept is promising for use in polymer analysis. Direct or indirect coupling with FID seems an interesting approach for universal quantification. There is currently no straightforward hyphenation method to LC however, severely limiting its current applicability. There are however some interesting developments ongoing.

### 3.13 Miscellaneous

This section features a variety of detectors that have not (yet) seen many routine applications in polymer analysis or LC in general either due to not being commercialized, being niche or to still being in development.

#### 3.13.1 Inductively coupled plasma‐mass spectrometry

Inductively coupled plasma‐MS (ICP‐MS) is a method that is mostly used in elemental speciation studies rather that polymer analysis [44]. In ICP‐MS, the analyte is fully atomized using an ICP followed by detection by MS. The detector offers the possibility to selectively monitor the elution of a variety of species of atoms over the elution profile [44]. ICP‐MS has currently seen little application in polymer characterization. It was applied to the quantification of polybrominated diphenyl ethers flame retardants in polymer samples by Mingwu et al. [140]. The method was able to quantify the injected amount of polybrominated diphenyl ethers to 1.18–1.51 ng on column. ICP‐MS is an interesting detection option for polymer analysis and could be applied to selectively monitor a specific atom in the polymer across a separation.

#### 3.13.2 Radioactivity detection

Radioactivity detectors detect radioactive compounds selectively, typically by means of a scintillation agent that emits light when exited by radioactive decay [34]. Radio activity detectors offer some attractive features such as a high sensitivity (pg) and good linear dynamic range (10^4^, Table 1). The applicability of radioactivity detection to polymer analysis is severely limited since it would require radioactive labeling, limiting it to its current main application area, metabolome studies [34, 141].

#### 3.13.3 Electrochemical detection

There is wide variety of electrochemical detectors (not including conductivity detection, described in Section 3.12) such as potentiometric, amperometric, coulometric, and voltammetric detectors, as well as a variety of operation modes and electrodes [142, 143]. This review will not cover these electrochemical detectors exhaustively, but instead focus on their potential application to polymer LC. In general, these detectors measure interactions at the junction of the electrode and the eluent. A typical example is the oxidation of carbohydrates at Ni‐electrodes under alkaline conditions, which can be detected by constant potential amperometric detection. Overall, electrochemical detectors are quite sensitive (ng‐fg) and feature a good linear range (10^5^, Table 1). Since most synthetic polymers are not electroactive, electrochemical detectors find little application in polymer analysis. Lloyd et al. demonstrated the application of electrochemical detection in SEC of nitrocellulose samples of various molecular weights achieving pg sensitivity [144]. Overall, electrochemical detectors are rarely applied in polymer LC, as many eluents used in polymer LC are not conductive and most polymers are not electroactive.

#### 3.13.4 Acoustic flame detector

The acoustic flame detector, proposed by Thurbide et al. as a universal detector in LC, functions by monitoring the oscillation of an oxygen hydrogen flame [41, 145]. The LC effluent is sprayed in the flame; carbon containing analytes affect the burn rate and thus the oscillation of the flame creating a signal. While the detector seemed quite promising in terms of sensitivity (ng) and linear range (∼10^3^), it was never commercially produced.

#### 3.13.5 Chemiluminescent nitrogen detection

The chemiluminescent nitrogen detector is a nitrogen‐specific detector. Analytes are oxidized under high temperatures causing nitrogen containing compounds to produce NO, which is converted to exited NO_2_ by reaction with ozone. This reaction is chemiluminescent, and the produced light is detected resulting in a detector response. This detector has been applied in both GC and LC [146]. Besides the chemiluminescent nitrogen detector, there is also a sulfur selective chemiluminescence detector, since it is even less applied in LC it will not be further covered [147]. The chemiluminescent nitrogen detector has been applied to SEC analysis of polyacrylamides in combination with RID, which allowed monitoring of the chemical composition over the MWD [148]. Due to being very niche the detector has seen little application in polymer analysis, but it might be an interesting detector for polyamide analysis. It is however too specific to be widely applied as a concentration sensitive detector.

#### 3.13.6 Density detection

The density detector, based on the mechanical oscillator principle, measures the density of the eluent over a chromatographic analysis [39]. The detector features an oscillating glass tube through which the eluent flows, and the period of the oscillation is dependent on the density of the eluent. The elution of an analyte changes the density and thus the period of the tube changes giving rise to a response. Trathnigg et al. provided much work on its application in polymer LC [56, 149, 150, 151]. The detector is unfortunately no longer commercially available. Son et al. more recently showed a novel miniaturized density detector based on suspended microchannel resonators, which supposedly has fg level sensitivity and a linear range of 10^6^ [152]. The detector was demonstrated on a SEC chromatogram of several PEG standards. Based on the work by Trathnigg et al. density detection yields a differential response for different polymers, thus yielding it unsuited as a universal response detector. Furthermore, it can only be used in isocratic mode [2]. This thus limits its applicability showing the same drawbacks as the commonly used RID.

#### 3.13.7 Magneto‐optical rotation detection

Another more recently proposed universal detector is the magneto‐optical rotation detector [153, 154]. This detector relies on the so‐called Faraday effect, which describes the interaction of polarized light with substances in a magnetic field. If the magnetic field is parallel to the light beam, the polarized light rotates based on the substance it passes through. This method is similar to optical activity measurements but requires no optical asymmetry and can thus be applied as a universal detector. Kawazumi et al. demonstrated its use in a SEC separation of polyethylene glycol yielding clearly detectable peaks, and the sensitivity was in the order of micrograms [154]. While an interesting alternative to RID, the response factor of these detectors is highly compound specific, thus rendering it unsuited as a universal concentration detector [153].

## 4 MULTIDETECTOR SETUPS

In polymer LC, often a combination of detectors is applied. This can be done to answer a variety of research questions and provides insights in different aspects of the sample depending on the exact combination of detectors. A number of common detector combinations will be reviewed mainly focusing on the determination of the chemical composition and the determination of the degree of branching.

### 4.1 Chemical composition

If a polymers composition changes across the MWD, it is called heterogeneous; this influences the functional properties of a polymer and is thus of interest. A combination of detectors with different selectivities can be applied to determine the chemical composition of a polymer across an elution profile and thus the heterogeneity.

**FIGURE 5:**
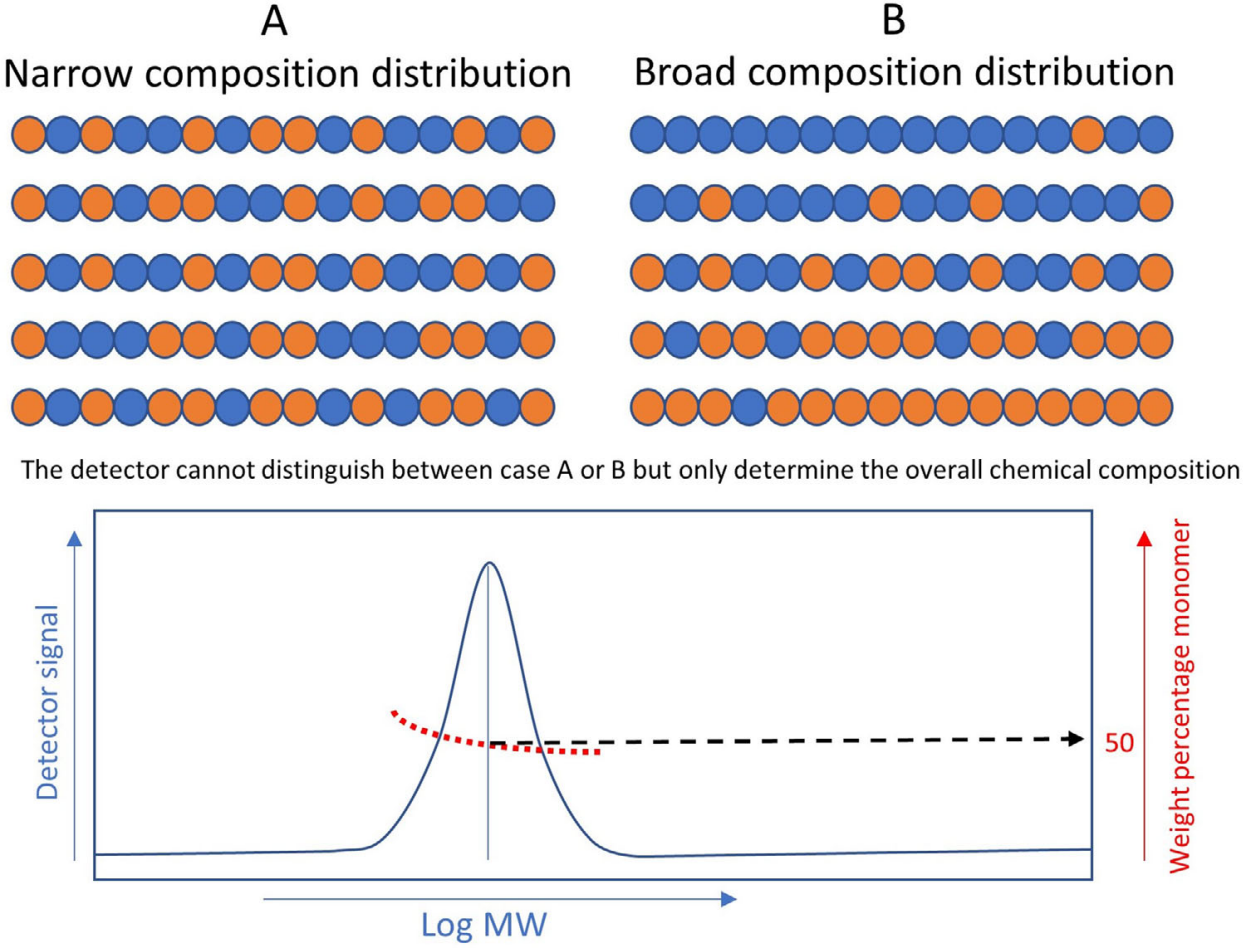
The detector measures the average chemical composition at a given elution point rather that the CCD

Detectors that yield chemical information are mainly applied in SEC to obtain an insight in changes of the average chemical composition (red line Figure 5) of a polymer as a function of the MWD (Figure 5). It should be highlighted that multidetector approaches only determine the average composition at a certain elution volume and not the CCD. For example, it is impossible to distinguish between two copolymers with a narrow and broad CCD with the same MW based on the determined chemical composition with a multidetector setup, as illustrated in Figure 5. To investigate the CCD, separation methods such as LAC [12, 155] or comprehensive 2D‐LC can be applied [17, 156].

Cools et al. describes the use of a combination of CAD, UV/VIS, and MS to obtain chemical composition and quantitative information on a multifunctional acrylate raw material [9]. Since the CAD offered a relative universal response, it allowed for the quantitative assessment of eluting polymers while the MS yielded information on the chemical composition of the eluting polymers.

The coupling of NMR, IR detection and MS to SEC, is a long time topic of interest [8, 26]. NMR and IR detectors are technically able to fully resolve the chemical composition of a polymer sample over a separation, given that the instrument is sensitive enough and the monomer spectra can be resolved. These detectors are however often still coupled to a RID or another concentration sensitive detector in tandem to monitor the overall concentration of eluting polymer.

**FIGURE 6:**
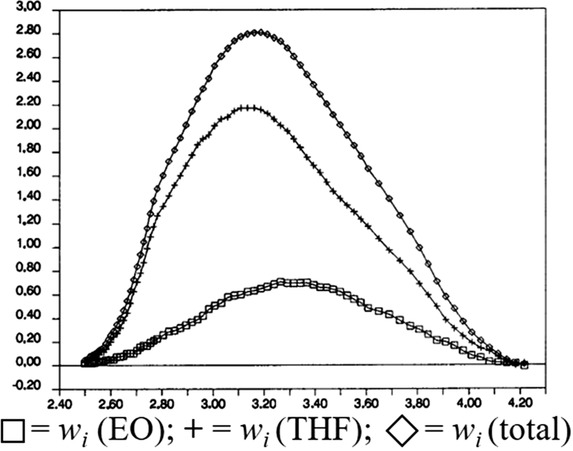
Example of the bulk chemical composition determination of a EO‐THF copolymer, *x*‐axis: log MW, *y*‐axis: response fractions of total signal. Reprinted from [150], copyright 1991, with permission from Elsevier

An alternative method to determine the heterogeneity of spectroscopic detectors is the application of multiple detectors with different response factors for the various monomers in the sample [55]. Various researchers have hyphenated multiple detectors, for example, RID‐ UV/VIS and RID‐density in tandem, to determine the chemical composition across a SEC separation [49, 50, 150]. Such methods generally determine the response factor for two homopolymers using a dual detection system. The bulk composition across the separation can be calculated from the difference in the response of the two detectors. This can, for instance, be achieved using a universal detector such as a RID and a selective detector such as a UV/VIS detector. Haidar et al. demonstrated this setup to determine the composition of poly(styrene‐co‐methyl methacrylate) as a function of MW [50]. Another example by Trathnigg et al. can be seen in Figure 6 where a density detector is applied in tandem with an RID to determine the chemical composition of a poly(ethylene oxide‐co‐tetrahydrofuran) copolymer across an SEC separation [150].

The density detector was also applied to determine the chemical composition in SEC of styrene‐buthadiene copolymers in combination with RID and showed good agreement with UV/VIS ‐RID results, typically yielding less than 1% difference in the determined average composition [149]. Hiller et al. expanded on this approach and analyzed a blend of poly(methyl methacrylate)/polystyrene/polyisoprene with RID and UV/VIS at different wavelengths [55]. Since each monomer featured a different response factor for different detectors, the overall composition could be calculated across an MW trace yielded from SEC. Furthermore, it was shown that not just the slope of the response function, but also the intercept could improve the accuracy of the calculated mass fractions.

### 4.2 Branching

There are a variety of detectors that can be hyphenated to study the molecular architecture, often as a function of the molecular weight of the polymer. Branching analysis is not directly related to quantitative detection but it is widely applied and heavily reliant on accurate quantitative information and thus covered in this review.

**FIGURE 7:**
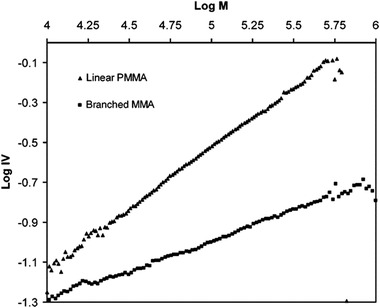
Mark–Houwink plot constructed by plotting the log M versus the log of the intrinsic viscosity. This plot allows for a clear distinction between linear and branched poly(methyl methacrylate), reprinted with permission from [78]. Copyright 2005, American Chemical Society

Besides universal calibration (see Section 3.10), multidetector setups are often applied to study the degree of branching. From a detector perspective, the determination of branching is challenging to investigate as there is chemically little difference in the molecules. To study branching, a combination of detectors is often applied in a so‐called "*triple‐detection"* setup, typically featuring a RID, a viscometer and a light‐scattering detector in tandem. By plotting [η] versus MW on a logarithmic scale, a Mark–Houwink plot is constructed. In this plot, the linear and branched varieties of the polymer will have different slopes allowing for a distinction between the two. Mitra et al. applied this method to determine the long‐chain branching of ethylene‐propylene‐diene rubber [157]. By constructing a Mark–Houwink plot, linear species could be distinguished from branched ones. Another example was reported by Saunders et al. who applied triple detection to characterize the degree of branching of poly(methyl methacrylate) [78] (Figure 7).

**FIGURE 8:**
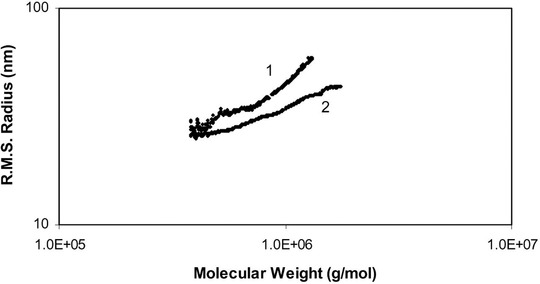
Plot of molecular weight versus root‐mean‐square radius of linear and branched polyvinyl acetate. Reprinted from permission from [77], copyright 2003, with permission from Elsevier

An alternative approach to investigate branching is by plotting the radius of gyration, which can be determined by MALS versus the MW, both on a log scale [22]. This method was first devised by Zimm and Stockmayer [158]. An example of this is featured in Figure 8, where the radius of gyration is plotted versus the logarithm of the molecular weight [77]. MALS is commonly applied to study a variety of aspects of polymers mainly focusing on branching as demonstrated by Barrera‐Rivera et al. [159] and Yu et al. [160], and conformational properties as shown by Teresa et al. [161].

Plüschke et al. applied quadruple‐detector SEC by addition of DLS to the more standard triple detection setup (RID, DV, and MALS) and the hydrodynamic radius (*R_H_*) could be determined, allowing for more in‐depth topological characterization of polyethylene [162]. Rowland et al. applied quintuple‐detector SEC, and by applying a combination of MALS, DLS, UV/VIS, RID, and DV, both the chemical composition and accurate information on the molecular size and conformation could be obtained [163]. The combination RID and UV/VIS detectors allowed for the determination of the specific refractive index increment of blends and copolymers of polyacrylamide and poly(N,N‐dimethylacrylamide) allowing for accurate determination of the concentration. Furthermore, using this information, a variety of molecular characteristics such as the intrinsic viscosity, the hydrodynamic radius, the viscometric radius, and the radius of gyration could be determined.

**FIGURE 9:**
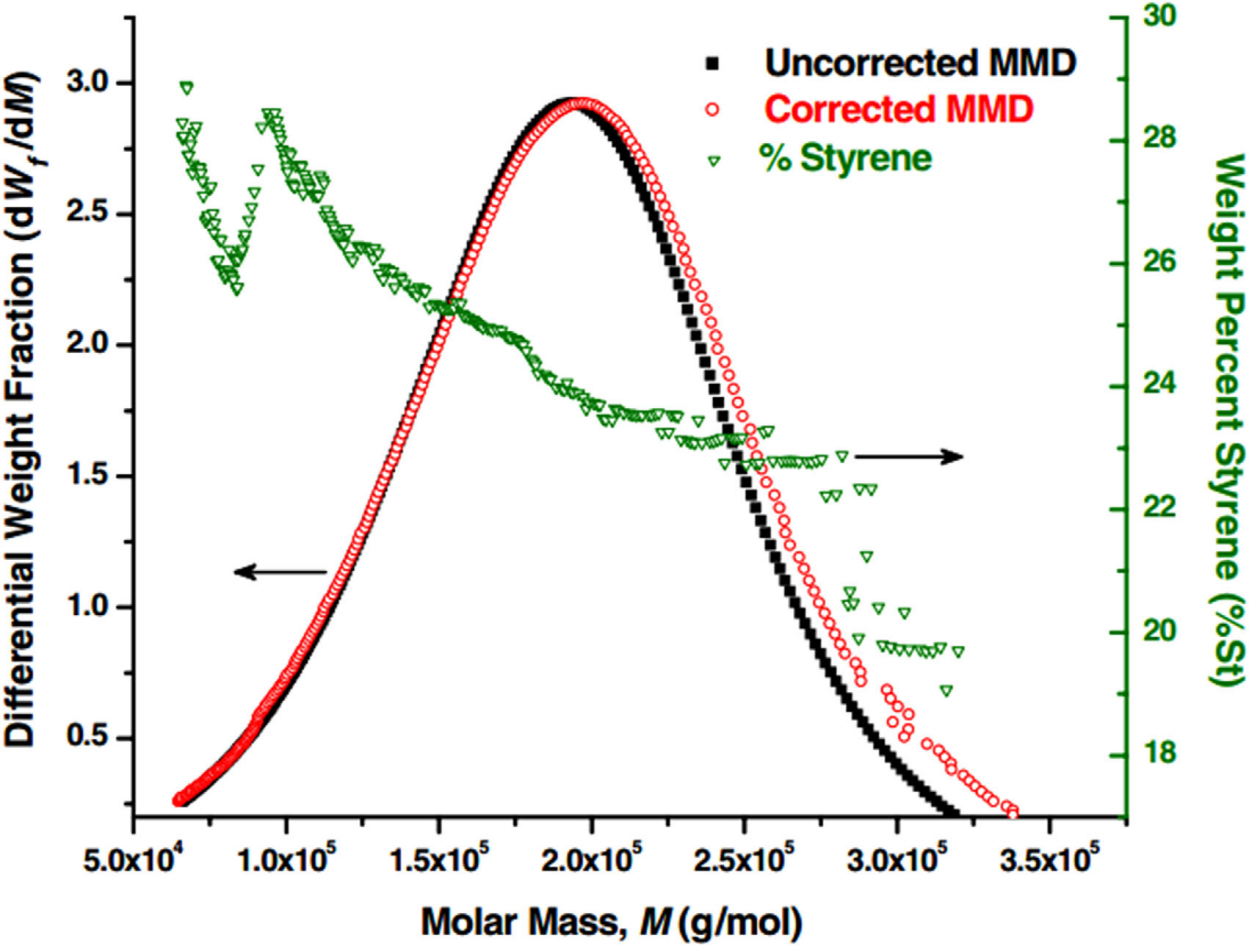
Representation of the corrected MW versus the uncorrected MW, with the weight percentage of styrene. Reprinted by permission from [50], copyright 2009 Springer‐Verlag

While multidetector combinations with LS detectors offer a wealth of information on the molecular structure, they do suffer from the drawback that accurate information on the dn/dc of the compound is required to accurately determine the MW from the LS signal. This is not trivial, in particular for heterogeneous copolymers, as it requires incremental determination of the *dn/dc*. An example of this on a polystyrene‐polymethylmethacrylate copolymer by Haidar et al. can be found in Figure 9, and the measured composition was determined by a combination of RID and UV/VIS detection [50].

While incremental determination is feasible for copolymers consisting of two monomers, it becomes increasingly difficult to apply as the number of monomers increases, which limits its application to the analysis of complex industrial polymers.

## 5 DISCUSSION AND CONCLUSION

The state‐of‐the‐art detectors and their application in polymer LC have been reviewed. One of the most important conditions for a quantitative LC detector's applicability to complex copolymers is that the detector has a uniform response factor, since many samples are heterogeneous in composition and thus feature different response factor across the separation. This renders detectors such as the RID but also other reviewed detectors such as the density detector, magnetic optical rotation based detectors, the acoustic flame detector, and novel mirroring resonator based RID detectors difficult to apply to complex samples as knowledge of possible changes in the response factor over the separation is required for accurate quantification.

Aerosol detectors such as the ELSD and CAD perform better regarding universal response than the RID, rendering them attractive options as concentration‐sensitive detectors in polymer LC. While requiring some data treatment to correct for nonlinear response factors, recent work using data pointwise linearization of the ELSD signal has shown promising results. The current main drawback seems the change in response factor caused by changes in eluent composition but solutions for this issue have also been proposed. NMR spectroscopy is another promising detector polymer LC, as it is intrinsically quantitative while offering a wealth of chemical information on the sample. Due to its discussed drawbacks such as high cost and low sensitivity, NMR spectroscopy is currently not routinely applicable. Recent work with lower field instruments is promising and might render the method more widely applicable. Another interesting approach for obtaining a predictable albeit not universal response is taking place in the form of the Solvere™, which yields a constant response per carbon atom in the analyte. Further developments in the field of coupling LC to pyrolysis‐GC are of interest as it would yield quantitative chemical information across a separation. The current bottleneck seems to be speeding up the GC separation enough to achieve a high enough data density on the separation.

Despite these interesting developments, the apparent limited magnitude of developments toward establishing quantitative detection techniques was surprising. Current detectors are too limited in their universal applicability toward the quantitation of polymers with varying compositional features. This renders these techniques inapplicable to contemporary complex polymers of which the chemical features are unknown. Consequently, accurate quantitative analysis in polymer separations is currently a significant bottleneck in the development of new sustainable materials. We are therefore convinced that this field requires significantly more attention.

## CONFLICT OF INTEREST

The authors have declared no conflict of interest.
